# The correlation between non-high-density lipoprotein cholesterol to high-density lipoprotein cholesterol ratio (NHHR) with non-alcoholic fatty liver disease: an analysis of the population-based NHANES (2017–2018)

**DOI:** 10.3389/fmed.2024.1477820

**Published:** 2024-11-08

**Authors:** Yuhao Yang, Shengxi Li, Zhenmei An, Shuangqing Li

**Affiliations:** ^1^General Practice Ward/International Medical Center Ward, General Practice Medical Center, West China Hospital, Sichuan University, Chengdu, China; ^2^Clinical Medical College, Southwest Medical University, Luzhou, China; ^3^Department of Endocrinology and Metabolism, West China Hospital, Sichuan University, Chengdu, China

**Keywords:** NHHR, NAFLD, NHANES, lipid metabolism, cross-sectional study

## Abstract

**Background/objective:**

Non-alcoholic fatty liver disease (NAFLD) encompasses a spectrum of liver disorders, from benign steatosis to more severe conditions like non-alcoholic steatohepatitis, with risks of progressing to fibrosis, cirrhosis, and hepatocellular carcinoma. The non-high-density lipoprotein cholesterol to high-density lipoprotein cholesterol ratio (NHHR) indicates lipid metabolic dysregulation and is associated with increased risks of various diseases. This study examines the relationship between NHHR and NAFLD to evaluate NHHR as a potential predictive biomarker for NAFLD.

**Methods:**

Data from the 2017–2018 National Health and Nutrition Examination Survey (NHANES) were used for cross-sectional analysis. After excluding individuals with incomplete data, hepatitis infections, heavy alcohol use, and those under 18, the study included 2,757 adults. The relationship between NHHR and NAFLD was analyzed using multivariable logistic regression, including subgroup analysis and interaction testing.

**Results:**

Among the 2,757 participants (mean age 49.91 years), 44.9% had NAFLD. NHHR showed a significant positive association with NAFLD, with an unadjusted odds ratio (OR) of 1.71 and a fully adjusted OR of 1.45. Quartile analysis revealed a 228% higher prevalence of NAFLD in the highest NHHR quartile, with an OR of 3.28. This positive association was consistent across various subgroups.

**Conclusion:**

Our findings suggest that elevated NHHR is positively correlated with the prevalence of NAFLD and possesses predictive value. We recommend that future research validate the clinical utility of NHHR, particularly for early detection of high-risk individuals and guiding personalized interventions.

## Introduction

Non-alcoholic fatty liver disease (NAFLD) includes a variety of liver diseases, such as non-alcoholic fatty liver, non-alcoholic steatohepatitis, and others, each with varying degrees of progression and liver damage (1). Over time, NAFLD can result in fibrosis, which may ultimately advance to cirrhosis and hepatocellular carcinoma (2). There is already evidence that NAFLD contributes significantly to liver disease worldwide, and it is expected to become the main cause of end-stage liver disease in the coming decades. The disease affects adults and children alike (3). The NAFLD incidence is believed to be about 30–40% in males and 15–20% in females (4). In addition, NAFLD is significantly linked to other comorbidities such as metabolic disease (5), cardiovascular diseases (6), obstructive sleep apnea (7), colorectal cancer (8), polycystic ovarian syndrome (9), chronic kidney disease (10), and osteoporosis (11).

Current research posits that hepatic lipid accumulation is integral to the pathogenesis of NAFLD (12). Dysregulation of lipid metabolism can induce stress in hepatocytes (13), activation of inflammasomes (14), and apoptotic cell death (15). Additionally, it can also contribute to inflammation, regeneration of tissues, and the formation of fibrous tissue (16). Prior research has shown a significant correlation between many lipid measures, including low-density lipoprotein cholesterol (LDL-C) (17), high-density lipoprotein cholesterol (HDL-C) (18), and residual cholesterol (19), and the development of NAFLD. The NHHR represents the equilibrium between HDL-C and non-HDL-C and functions as an innovative marker of atherogenic lipid composition. Elevated NHHR levels have been correlated with heightened risks of multiple diseases, including type 2 diabetes (20), hyperuricemia (21), nephrolithiasis (22), and osteoporosis (21). The body of research indicates that NHHR may serve as a valuable predictor of metabolic-related diseases (20). Nevertheless, there remains a paucity of research investigating the association between NHHR and NAFLD.

As a result, we aim to perform a cross-sectional study of U.S. adults using NHANES data. The purpose of this study was to elucidate the association between NHHR and NAFLD, thus assessing NHHR as a predictive biomarker. It may be possible to develop preventative and therapeutic measures based on these findings.

## Method

### Study participants

This national survey conducted by the National Center for Health Statistics (NCHS) in 2017–2018 was utilized to evaluate the nutritional and health status of U.S. residents. Initiated in 1971, this study used a sophisticated multistage probability design to obtain a sample representing non-institutionalized U.S. residents. The NHANES is a nationwide survey focusing on people who do not live in institutions and live in communities within the U.S. There is comprehensive survey material and procedural guides available at https://www.cdc.gov/nchs/nhanes/index.htm. It utilizes sample strategies that include many stages, stratification, and complicated probability methods, with intentional oversampling of specific subpopulations, including racial/minority groups and older adults (23). Because the data evaluated in this research was de-identified, our local institutional review board did not need to assess it.

**Figure 1:**
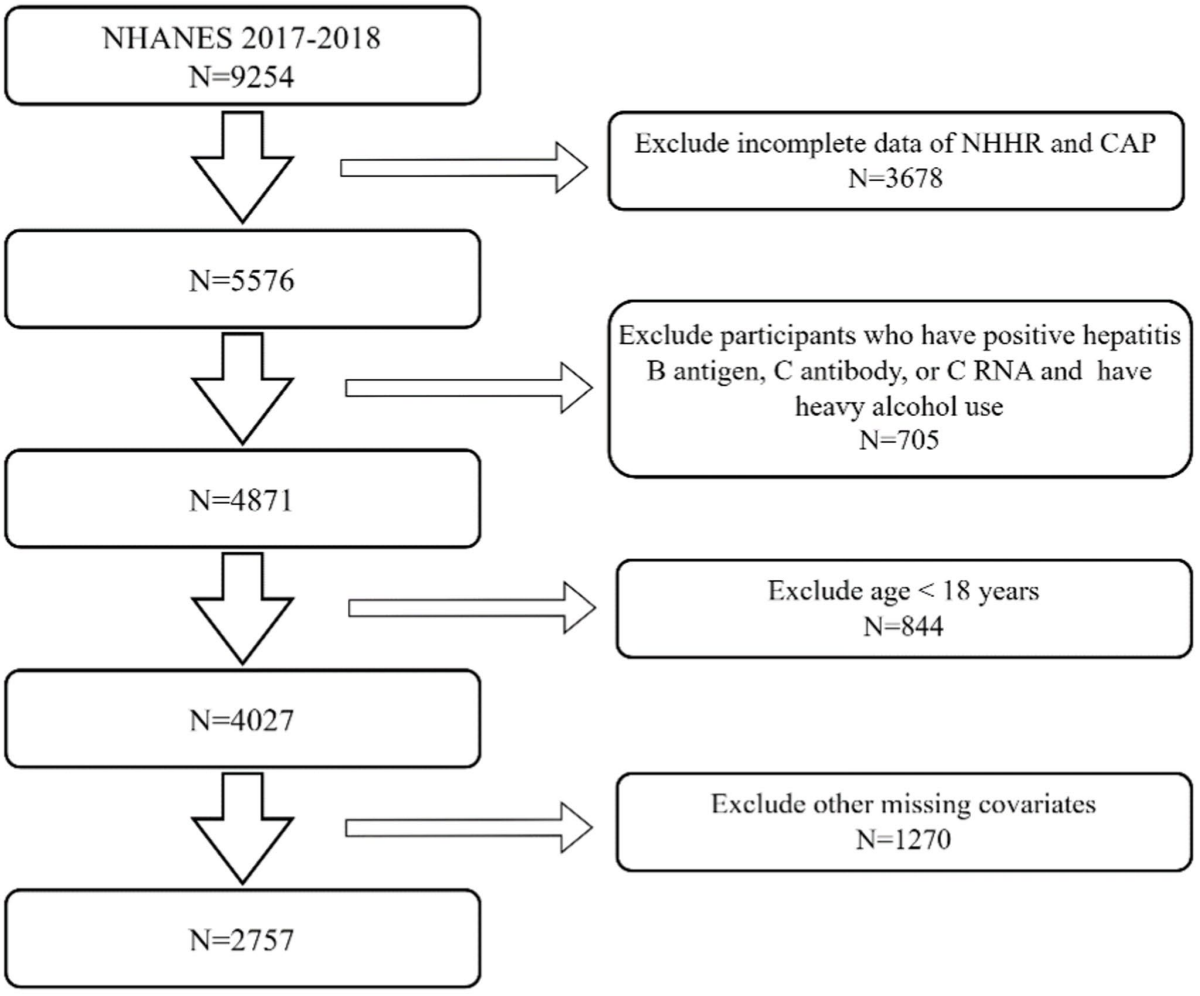
Flowchart of the sample selection from NHANES 2017–2018.

The National Health and Health Administration sampled 9,254 participants from the NHANES 2017–2018 dataset. The exclusion criteria for our research were as follows: (I) The data on total cholesterol (TC), HDL-C, and NHHR is incomplete for 3,678 people. (II) There are 705 participants who have positive hepatitis B antigen, C antibody, or C RNA and also have heavy alcohol use (consuming 4, 5, or more drinks per day). (III) There are 844 individuals who are below 18 years of age. (IV) There are 1,270 individuals who have missing data for body mass index (BMI), poverty-to-income ratio (PIR), educational level, hypertension, diabetes, hyperlipidemia, drinking status, and smoking status. After excluding individuals who did not fit the specified criteria or had incomplete data, typically, 2,757 participants were chosen as participants for this research (Figure 1).

### Outcome variables

The controlled attenuation parameter (CAP) and liver stiffness measurements (LSM) were chosen as outcome variables to examine the existence of hepatic steatosis and liver fibrosis, respectively (24). The NHANES team performed assessments on individuals utilizing Vibration Controlled Transient Elastography (VCTE) with the FibroScan-equipped model 502 V2 Touch (25). Recent research found that CA *p-*values ≥274 dB/m could be used to identify NAFLD because of their high sensitivity of 90% in identifying all levels of hepatic steatosis (24).

### Exposure assessment

The NHHR is ascertained by first subtracting the HDL-C from the TC and subsequently calculating the ratio of this difference to the HDL-C (26). Measurements are conducted using the Beckman Coulter UniCel^®^ DxC 800 Synchron chemistry analyzer, which employs a timed endpoint method to evaluate various lipid parameters. Specifically, for NHHR determination, both TC and HDL-C are quantified, and non-HDL cholesterol is derived from these values. The methodology involves enzymatic reactions and monitoring absorbance changes at designated wavelengths, thereby ensuring high accuracy and reliability in the measurements.

### Covariates

In our research, we incorporated a range of covariates that may have an impact on the outcome. The covariates included variables encompassing race, age, gender, PIR, educational level, BMI, smoking behaviors, alcohol intake, the existence of hyperlipidemia, diabetes, and hypertension. The racial and ethnic classifications examined were Non-Hispanic Black, Non-Hispanic White, Mexican American, different Hispanic, and other Race. To determine a person’s BMI, divide their weight in kilograms by their height in meters squared. Following this calculation, individuals are classified into weight categories based on their body fat percentage: less than 18 kg/m^2^ as underweight, 18–25 kg/m^2^ as normal weight, 25–29.9 kg/m^2^ as overweight, and 30 kg/m^2^ as obese. The classification of educational attainment was classified into five categories: below high school, incomplete high school, high school completion, post-secondary education, and higher education degree. The smoking status was categorized as either never smoking (also known as having smoked <100 tobacco sticks in one’s lifetime) or presently smoking (having smoked 100 or more tobacco sticks in one’s lifetime). Alcohol intake was classified as non-drinking if the individual had drunk alcohol less than 12 occasions in the preceding year and as drinking if they drank alcohol 12 or more occasions in the preceding year. Data on the incidence of hypertension, hyperlipidemia, and diabetes among people was gathered by self-reported questionnaires.

### Statistical analysis

Continuous variables were described using mean values plus or minus standard error (SE), while categorical characteristics were presented as proportions. We evaluated variations among people categorized by NHHR quantiles using Student’s t-tests for continuous variables and chi-square testing for categorical variables. To determine the association between NHHR (as both a continuous variable and in quartiles) and NAFLD, we employed multivariable logistic regression while taking into account various covariates. Three models were employed: The first model was used without any adjustments, the second was modified for age, gender, and race, and the third was expanded to include all covariates relevant to the study. The NHHR-NAFLD correlation was estimated by computing odds ratios (ORs) with 95% confidence intervals (CIs). To further explore this association, a comprehensive methodological approach was employed, including variable adjustment and smooth curve fitting to identify potential non-linear relationships. Subsequent subgroup analyses were performed to investigate the NHHR-NAFLD connection across different demographic and clinical subgroups, including gender, age, BMI, hypertension, hyperlipidemia, and diabetes status. We performed the statistical analysis and graphics computing using R software (version 4.1.0) and EmpowerStats (version 2.0).

## Results

### Baseline characteristics of participants

Our analysis included 2,757 participants with a mean age of 49.91 ± 17.50 years; 46.25% were men, and 53.75% were women. NAFLD was found in 44.90% of participants. Table 1 shows the baseline characteristics by NAFLD status. Significant variations in age, gender, race, educational level, BMI, NHHR, TC, HDL-C, smoking status hypertension, diabetes, and hyperlipidemia (*p* < 0.05) were observed between participants with and without NAFLD. The difference in PIR and drinking status did not show statistical significance (*p* > 0.05). NAFLD patients were generally older, predominantly male, with higher BMI (obese), elevated NHHR, and a greater prevalence of diabetes, hyperlipidemia, hypertension, and smoking compared to those without NAFLD.

**Table 1:** Baseline characteristics of participants according to NAFLD status.

Characteristic	Non-NAFLD	NAFLD	*P-*value
	*N* = 1,519	*N* = 1,238	
Age (years)	47.65 ± 18.31	52.67 ± 16.03	<0.001
Gender, %			<0.001
Male	623 (41.01%)	652 (52.67%)	
Female	896 (58.99%)	586 (47.33%)	
Race, %			<0.001
Mexican American	158 (10.40%)	227 (18.34%)	
Different Hispanic	133 (8.76%)	110 (8.89%)	
Non-Hispanic White	572 (37.66%)	473 (38.21%)	
Non-Hispanic Black	378 (24.88%)	225 (18.17%)	
Other Race	278 (18.30%)	203 (16.40%)	
BMI, % (kg/m^2^)			<0.001
<18.5	34 (2.24%)	0 (0.00%)	
18.5–24.9	569 (37.46%)	88 (7.11%)	
25.0–29.9	519 (34.17%)	376 (30.37%)	
≥30	397 (26.14%)	774 (62.52%)	
Education level,%			<0.001
Below high school	80 (5.27%)	86 (6.95%)	
Incomplete high school	133 (8.76%)	126 (10.18%)	
High school completion	337 (22.19%)	307 (24.80%)	
Post-secondary education	518 (34.10%)	435 (35.14%)	
Higher education degree	451 (29.69%)	284 (22.94%)	
PIR	2.72 ± 1.62	2.67 ± 1.58	0.423
Hypertension, %			<0.001
Yes	403 (26.53%)	576 (46.53%)	
No	1,116 (73.47%)	662 (53.47%)	
Hyperlipidemia, %			<0.001
Yes	437 (28.77%)	522 (42.16%)	
No	1,082 (71.23%)	716 (57.84%)	
Diabetes, %			<0.001
Yes	137 (9.02%)	270 (21.81%)	
No	1,382 (90.98%)	968 (78.19%)	
Smoking status,%			0.006
Yes	137 (9.02%)	270 (21.81%)	
No	1,382 (90.98%)	968 (78.19%)	
Drinking status, %			0.216
Yes	649 (42.73%)	500 (40.39%)	
No	870 (57.27%)	738 (59.61%)	
TC (mg/dL)	186.11 ± 39.93	192.17 ± 41.83	<0.001
HDL-C (mg/dL)	57.57 ± 15.24	48.77 ± 14.70	<0.001
NHHR	2.42 ± 1.08	3.22 ± 1.43	<0.001

Based on the NHHR quartiles, Table 2 shows the clinical characteristics of patients. The mean NHHR was 2.78 ± 1.31, with NHHR quartiles ranging from 0.28–1.84 (Q1), 1.85–2.54 (Q2), 2.55–3.44 (Q3), to 3.45–12.32 (Q4). Significant differences were found among these quartiles in gender, race, BMI, PIR, educational level, drinking status, smoking status, hyperlipidemia, and hypertension (all *p* < 0.05). Individuals in the upper quartiles of NHHR were shown to have a greater likelihood of being male, obese, having lower income, and having an elevated prevalence of hypertension, hyperlipidemia, and smoking contrasted with those in the lower quartiles of NHHR (*p* < 0.05).

**Table 2:** Baseline characteristics of participants according to the NHHR’s quartile.

Characteristic	Quartiles of NHHR				*p*-value
	Q1 (*N* = 685)	Q2 (*N* = 687)	Q3 (*N* = 694)	Q4 (*N* = 691)	
Age (years)	49.75 ± 19.68	49.54 ± 18.23	50.74 ± 16.46	49.58 ± 15.35	0.49
Gender,%					<0.001
Male	226 (32.99%)	263 (38.28%)	355 (51.15%)	431 (62.37%)	
Female	459 (67.01%)	424 (61.72%)	339 (48.85%)	260 (37.63%)	
Race, %					<0.001
Mexican American	73 (10.66%)	74 (10.77%)	117 (16.86%)	121 (17.51%)	
Different Hispanic	48 (7.01%)	59 (8.59%)	58 (8.36%)	78 (11.29%)	
Non-Hispanic White	259 (37.81%)	272 (39.59%)	259 (37.32%)	255 (36.90%)	
Non-Hispanic Black	195 (28.47%)	162 (23.58%)	141 (20.32%)	105 (15.20%)	
Other Race	110 (16.06%)	120 (17.47%)	119 (17.15%)	132 (19.10%)	
BMI, % (kg/m^2^)					<0.001
<18.5	21 (3.07%)	6 (0.87%)	4 (0.58%)	3 (0.43%)	
18.5–24.9	285 (41.61%)	187 (27.22%)	113 (16.28%)	72 (10.42%)	
25.0–29.9	202 (29.49%)	210 (30.57%)	250 (36.02%)	233 (33.72%)	
≥30	177 (25.84%)	284 (41.34%)	327 (47.12%)	383 (55.43%)	
Education level, %					<0.001
Below high school	29 (4.23%)	31 (4.51%)	41 (5.91%)	65 (9.41%)	
Incomplete high school	58 (8.47%)	50 (7.28%)	70 (10.09%)	81 (11.72%)	
High school completion	176 (25.69%)	147 (21.40%)	165 (23.78%)	156 (22.58%)	
Post-secondary education	220 (32.12%)	252 (36.68%)	253 (36.46%)	228 (33.00%)	
Higher education degree	202 (29.49%)	207 (30.13%)	165 (23.78%)	161 (23.30%)	
PIR	2.80 ± 1.62	2.81 ± 1.59	2.63 ± 1.61	2.55 ± 1.58	0.004
Hypertension status, %					0.043
Yes	219 (31.97%)	236 (34.35%)	255 (36.74%)	269 (38.93%)	
No	466 (68.03%)	451 (65.65%)	439 (63.26%)	422 (61.07%)	
Hyperlipidemia, %					<0.001
Yes	185 (27.01%)	222 (32.31%)	236 (34.01%)	316 (45.73%)	
No	500 (72.99%)	465 (67.69%)	458 (65.99%)	375 (54.27%)	
Diabetes status, %					0.623
Yes	101 (14.74%)	95 (13.83%)	99 (14.27%)	112 (16.21%)	
No	584 (85.26%)	592 (86.17%)	595 (85.73%)	579 (83.79%)	
Smoking status, %					<0.001
Yes	237 (34.60%)	260 (37.85%)	286 (41.21%)	319 (46.16%)	
No	448 (65.40%)	427 (62.15%)	408 (58.79%)	372 (53.84%)	
Drinking status, %					<0.001
Yes	330 (48.18%)	281 (40.90%)	287 (41.35%)	251 (36.32%)	
No	355 (51.82%)	406 (59.10%)	407 (58.65%)	440 (63.68%)	

### Association between NHHR and NAFLD

Table 3 displays the findings of a generalized linear regression model that investigates the correlation between NHHR and NAFLD. The original unadjusted model manifested a positive connection between NHHR and NAFLD (OR = 1.71, 95% CI: 1.59, 1.83). This association remained significant after controlling for different demographic and health-related variables involving gender, age, race, BMI, educational level, drinking, smoking, diabetes, hyperlipidemia, and hypertension (OR = 1.45, 95% CI: 1.34, 1.57). These findings suggest that for each unit increase in NHHR, there is a 45% higher likelihood of NAFLD prevalence. When NHHR was examined as a categorical variable in quartiles, persons in the largest NHHR quartile exhibited a 228% higher prevalence of NAFLD in comparison to those in the smallest quartile, emerging statistical significance (OR = 3.28, 95% CI: 2.47, 4.34). Similar positive correlations were noted in the second-highest (OR = 1.46, 95% CI: 1.11, 1.92) and third-highest (OR = 2.14, 95% CI: 1.63, 2.80) NHHR quartiles.

**Table 3:** Multiple logistic regression associations of NHHR with NAFLD in adults.

NHHR	Model 1 OR (95% CI) *P-*value	Model 2 OR (95% CI) *P-*value	Model 3 OR (95% CI) *P-*value
Continuous	1.71 (1.59, 1.83) <0.0001	1.68 (1.57, 1.81) <0.0001	1.45 (1.34, 1.57) <0.0001
Categories			
Q1	1.0(ref)	1.0(ref)	1.0(ref)
Q2	1.80 (1.43, 2.28) <0.0001	1.83 (1.45, 2.33) <0.0001	1.46 (1.11, 1.92) 0.0063
Q3	3.12 (2.49, 3.92) <0.0001	2.96 (2.34, 3.74) <0.0001	2.14 (1.63, 2.80) <0.0001
Q4	5.64 (4.47, 7.12) <0.0001	5.39 (4.23, 6.88) <0.0001	3.28 (2.47, 4.34) <0.0001

### Non-linear relationship between NHHR and NAFLD

**Figure 2:**
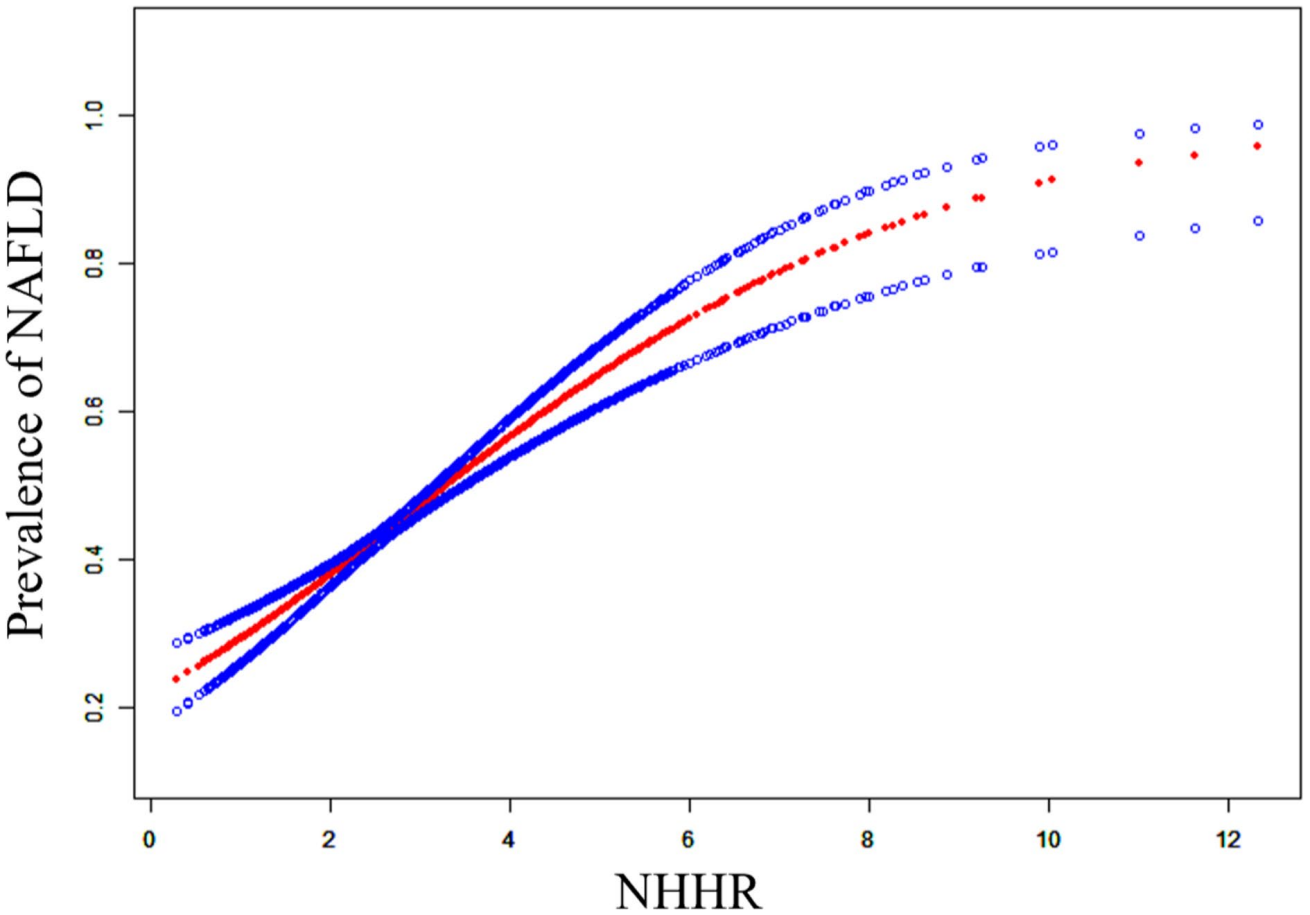
Smooth curve fitting for NHHR and NAFLD.

To determine the possible correlation between NHHR and NAFLD, we used a curve-fitting technique to identify a non-linear association between the two variables (Figure 2). We further identified the inflection point at 2.60. When NHHR is below 2.60, for each unit increase in NHHR, the prevalence of NAFLD increases by 69% (OR: 1.69, 95% CI: 1.36, 2.10). When NHHR exceeds 2.60, for each unit elevation in NHHR, the NAFLD prevalence increases by 36% (OR: 1.36, 95% CI: 1.22, 1.53).

### NHHR-NAFLD relationship subgroup analysis

**Figure 3:**
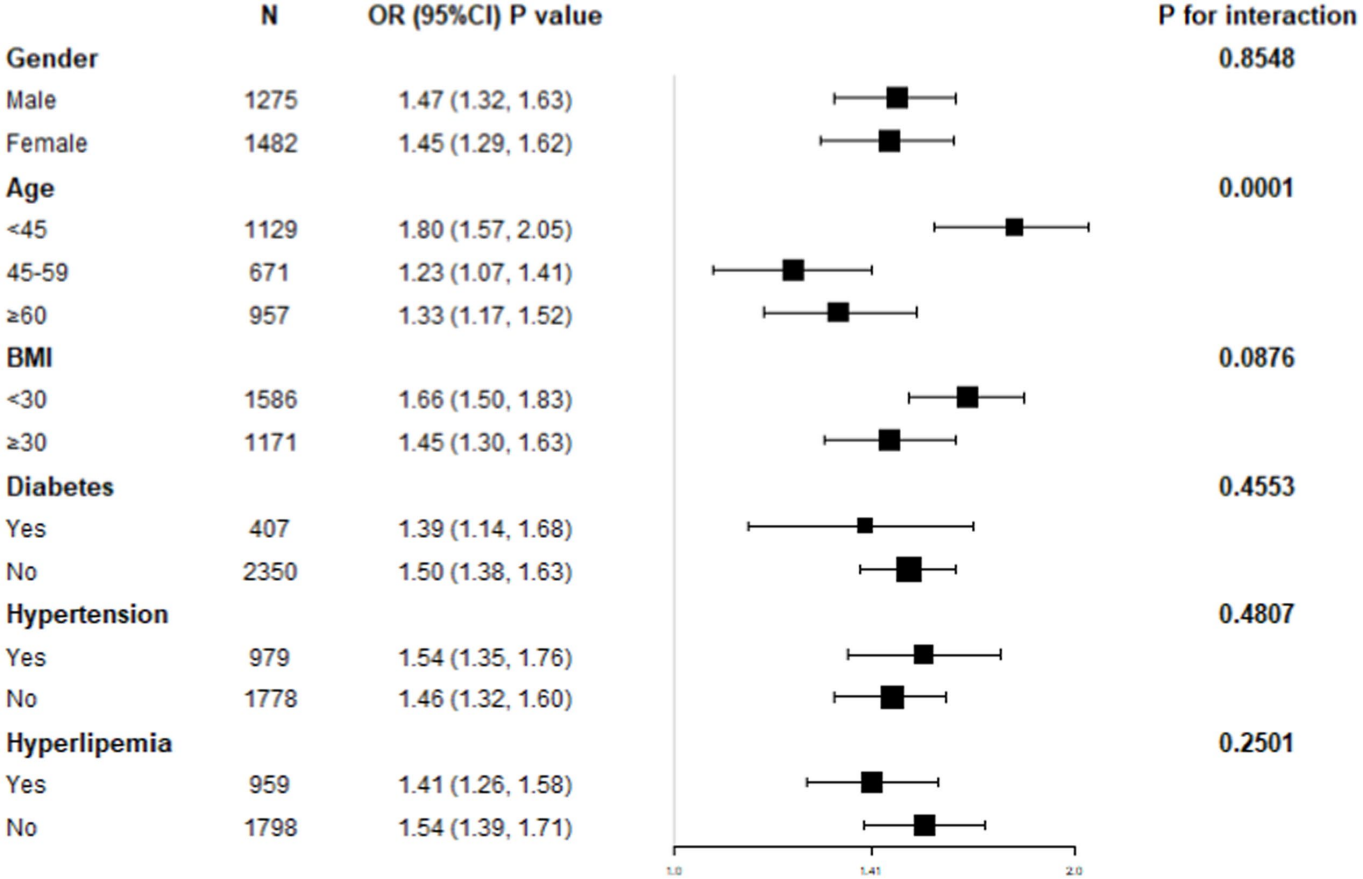
Subgroup analysis for the association between NHHR and NAFLD.

A subgroup study was performed to ascertain the association uniformity between NHHR and NAFLD across different demographic cohorts (Figure 3). Age stratification demonstrated a statistically significant and independent positive connection between NHHR and NAFLD (*p* for interaction <0.05). In contrast, the stratifications according to gender, BMI, diabetes, hypertension, and hyperlipidemia status did not possess a significant impact on the positive relationship between NHHR and NAFLD (*p* for interaction >0.05).

## Discussion

A group of 2,757 individuals, carefully selected to represent the entire population of US adults, took part in the study. It was found that NHHR is associated with a higher incidence of NAFLD. Furthermore, a non-linear correlation between NHHR and NAFLD was discovered. The point of inflection was determined to be at a value of 2.60. Analyzed subgroups stratified by gender, age, BMI, hypertension, and diabetes and conducted interaction testing, demonstrated a similar positive connection across different populations.

This research is the first exploration of the relationship between NHHR and NAFLD. Due to the increasing occurrence of lipid metabolism disorders and NAFLD in adults, various lipid components have been studied concerning NAFLD in several previous studies. For instance, a study from the NAGALA cohort, which included 14,251 subjects, showed that multiple lipid parameters have significant mediating effects between BMI and NAFLD, suggesting that relevant composite lipid parameters should be monitored when intervening in BMI to prevent or treat NAFLD (18). Another cohort study from a region in China, which included 12,126 participants, utilized the combined index of HDL-C and gamma-glutamyl transferase (GGT) to explore their relationship with NAFLD. According to this study, the GGT/HDL-C ratio predicts NAFLD more accurately than HDL-C alone in early (1–3 years) stages of the disease, further confirming the accuracy and advantage of non-traditional lipid indices over traditional ones in assessing NAFLD (27). Additionally, a prospective study involving 213 subjects in Israel over approximately 7 years of follow-up found that the novel composite lipid parameter non-HDL-C and the traditional lipid parameter LDL-C are significant independent predictors of NAFLD, with non-HDL-C being the stronger predictor (28). An increasing body of literature supports studying the association between lipid profiles and NAFLD using new lipid characteristics.

An indicator of the non-HDL-C/HDL-C ratio, the NHHR is a newly developed method for measuring HDL-C levels, offering insights into the composition of atherogenic lipids. Empirical investigations have shown that NHHR is significantly better than standard lipid indicators in assessing the risk of atherosclerosis (29). Furthermore, research by Wang et al. has identified NHHR as a more efficacious tool for evaluating the influence of lipid metabolism on hyperuricemia (30). Similarly, Tan et al. have suggested that NHHR may serve as a useful marker for estimating the likelihood of developing type 2 diabetes mellitus (20). Furthermore, researchers have employed the NHHR as a more precise lipid predictor for assessing the risk of cardiovascular events (29), kidney stones (22), and osteoporosis (21). Collectively, these studies indicate that NHHR may be used as a potent instrument for forecasting metabolic-related diseases.

Herein, we undertook a preliminary investigation into the relationship between NHHR and NAFLD, revealing a strong positive connection between NHHR levels and the incidence of NAFLD. These findings suggest that NHHR could be a valuable lipid marker for predicting NAFLD.

A variety of liver conditions fall under the category of NAFLD, ranging from non-alcoholic fatty liver to non-alcoholic steatohepatitis, each characterized by varying degrees of liver injury and disease progression. NAFLD can lead to fibrosis, which may eventually cause cirrhosis and hepatocellular carcinoma (1). The original “two-hit hypothesis” posited that lipid accumulation in hepatocytes constitutes the “first hit, “whereas oxidative stress and other factors represent the “second hit” (31). However, recent evidence indicates that the pathogenesis of NAFLD is a multifactorial process, now referred to as the “multiple parallel hits hypothesis.” This modern model includes several elements that contribute to the condition, such as insulin resistance, genetic and epigenetic impacts, mitochondrial dysfunction, endoplasmic reticulum stress, gut microbiome dysbiosis, and chronic low-grade inflammation (32).

The mechanisms underlying the relationship between NHHR and NAFLD remain inadequately understood. Lipid metabolic imbalance, which forms the basis of NAFLD pathogenesis, induces cellular stress, activates the inflammasome, triggers apoptotic cell death, and promotes inflammation, tissue regeneration, and fibrosis (14–16). Given the pivotal role of lipid metabolic imbalance in NAFLD, we hypothesize that NHHR may influence NAFLD by mediating lipid metabolic dysregulation. Elevated levels of non-HDL-C, particularly LDL-C, and VLDL-C, can facilitate the accumulation of cholesterol and triglycerides, thereby increasing the risk of hepatic lipid deposition. In contrast, HDL-C helps prevent hepatic lipid deposition through reverse cholesterol transport and antioxidant and anti-inflammatory effects (33).

The development of NAFLD may also be influenced by NHHR through multiple oxidative stress pathways. Elevated NHHR signifies increased levels of non-HDL-C, particularly LDL-C, which is susceptible to oxidation, resulting in forming oxidized low-density lipoprotein (ox-LDL). Ox-LDL is highly atherogenic and can initiate oxidative stress responses, leading to hepatocyte injury and apoptosis (34). Moreover, HDL-C possesses antioxidant properties that mitigate lipid peroxidation and oxidative stress via reverse cholesterol transport and the activity of antioxidant enzymes (35). However, an elevated NHHR is indicative of reduced HDL-C levels, which compromises the body’s capacity to defend against antioxidants and makes the liver more susceptible to oxidative stress. This oxidative stress not only inflicts direct damage on hepatocytes but also activates Kupffer cells and hepatic stellate cells, releasing pro-inflammatory cytokines encompassing interleukin 6 (IL-6) and tumor necrosis factor-alpha (TNF-*α*). Consequently, this exacerbates hepatic inflammation and injury (36, 37). Moreover, empirical evidence has demonstrated a strong correlation between high NHHR and insulin resistance (38). We hypothesize that an elevated NHHR may contribute to insulin resistance, subsequently enhancing hepatic lipogenesis and inhibiting lipid catabolism, thereby facilitating hepatic steatosis (39). While this study has investigated the potential mechanisms connecting NHHR with NAFLD, these pathways are not yet fully understood and warrant further research.

Our study demonstrates a significant positive correlation between NHHR and NAFLD, potentially influenced by lipid metabolism dysregulation and oxidative stress pathways. However, this relationship may also be affected by certain unmeasured lifestyle factors and dietary patterns. Lifestyle factors, such as physical activity levels and sedentary behavior, as well as dietary habits, including high-fat and high-sugar diets, are crucial determinants of NAFLD that were not specifically included in our analysis.

For instance, previous studies have shown that low levels of physical activity are closely associated with a higher incidence of NAFLD, primarily due to insufficient energy expenditure, which leads to increased hepatic fat accumulation (40, 41). Additionally, high-fat and high-sugar diets can directly promote liver lipid accumulation and impact insulin sensitivity, potentially influencing NHHR levels and the progression of NAFLD (42). Therefore, lifestyle factors may play a moderating role in the impact of NHHR on NAFLD, and future studies should further explore these mechanisms. Considering that lifestyle interventions, such as increasing physical activity and improving dietary habits, have demonstrated positive effects on managing NAFLD, this suggests that in clinical practice, addressing these factors might reduce the risk of NAFLD in individuals with elevated NHHR, thereby improving intervention outcomes.

This study has several significant strengths. First, it is the first study globally to explore the relationship between NHHR and NAFLD, innovatively proposing the potential application of NHHR as a novel lipid marker for the early detection of NAFLD. This provides a new direction for the early identification of lipid metabolism-related diseases. Second, the study is based on the 2017–2018 National Health and Nutrition Examination Survey (NHANES) database, which is nationally representative and includes rich clinical and demographic information, enhancing the reliability and generalizability of our findings. Additionally, the study used rigorous multivariable regression analyses, adjusting for multiple confounders such as age, sex, and BMI, thereby reducing the impact of bias on the study results. However, this study has some limitations. First, as a cross-sectional study, we cannot infer causality between NHHR and NAFLD. While we proposed potential mechanisms, such as lipid metabolism dysregulation and oxidative stress pathways, experimental and longitudinal data are needed to confirm these mechanisms. Second, although we used vibration-controlled transient elastography (VCTE) as a non-invasive diagnostic tool, we did not compare it with other common NAFLD diagnostic methods, such as the Fatty Liver Index (FLI), Hepatic Steatosis Index (HSI), or imaging techniques like ultrasound and magnetic resonance elastography (MRE). Future studies incorporating these methods could help validate the applicability of NHHR. Additionally, the NHANES data is primarily based on the U.S. population, which may limit the generalizability of our findings to other populations. Differences in dietary habits, lifestyle, and genetic factors may influence the relationship between NHHR and NAFLD, so further studies in diverse populations are needed. Lastly, the NHANES dataset lacks information on lipid-lowering medication use, such as statins, which can impact LDL-C levels and, consequently, NHHR. This may introduce residual confounding, which future studies could address with more comprehensive datasets.

## Conclusion

We suggest that a high NHHR is positively correlated with the incidence of NAFLD and can be used to predict NAFLD. We hope that future research will confirm the usefulness of NHHR in clinical practice, especially in identifying individuals at high risk early on and in guiding personalized interventions to improve its role in preventing and treating NAFLD.

## Data Availability

Publicly available datasets were analyzed in this study. This data can be found: https://www.cdc.gov/nchs/nhanes/index.htm.
